# Identification of Neuropsychiatric Copy Number Variants in a Health Care System Population

**DOI:** 10.1001/jamapsychiatry.2020.2159

**Published:** 2020-07-22

**Authors:** Christa Lese Martin, Karen E. Wain, Matthew T. Oetjens, Kasia Tolwinski, Emily Palen, Abby Hare-Harris, Lukas Habegger, Evan K. Maxwell, Jeffrey G. Reid, Lauren Kasparson Walsh, Scott M. Myers, David H. Ledbetter

**Affiliations:** 1 Autism & Developmental Medicine Institute, Geisinger, Danville, Pennsylvania; 2 Biomedical Ethics Unit, McGill University, Montreal, Quebec, Canada; 3 Bloomsburg University, Bloomsburg, Pennsylvania; 4 Regeneron Genetics Center, Tarrytown, New York

## Abstract

**Importance:**

Population screening for medically relevant genomic variants that cause diseases such as hereditary cancer and cardiovascular disorders is increasing to facilitate early disease detection or prevention. Neuropsychiatric disorders (NPDs) are common, complex disorders with clear genetic causes; yet, access to genetic diagnosis is limited. We explored whether inclusion of NPD in population-based genomic screening programs is warranted by assessing 3 key factors: prevalence, penetrance, and personal utility.

**Objective:**

To evaluate the suitability of including pathogenic copy number variants (CNVs) associated with NPD in population screening by determining their prevalence and penetrance and exploring the personal utility of disclosing results.

**Design, Setting, and Participants:**

In this cohort study, the frequency of 31 NPD CNVs was determined in patient-participants via exome data. Associated clinical phenotypes were assessed using linked electronic health records. Nine CNVs were selected for disclosure by licensed genetic counselors, and participants’ psychosocial reactions were evaluated using a mixed-methods approach. A primarily adult population receiving medical care at Geisinger, a large integrated health care system in the United States with the only population-based genomic screening program approved for medically relevant results disclosure, was included. The cohort was identified from the Geisinger MyCode Community Health Initiative. Exome and linked electronic health record data were available for this cohort, which was recruited from February 2007 to April 2017. Data were collected for the qualitative analysis April 2017 through February 2018. Analysis began February 2018 and ended December 2019.

**Main Outcomes and Measures:**

The planned outcomes of this study include (1) prevalence estimate of NPD-associated CNVs in an unselected health care system population; (2) penetrance estimate of NPD diagnoses in CNV-positive individuals; and (3) qualitative themes that describe participants’ responses to receiving NPD-associated genomic results.

**Results:**

Of 90 595 participants with CNV data, a pathogenic CNV was identified in 708 (0.8%; 436 women [61.6%]; mean [SD] age, 50.04 [18.74] years). Seventy percent (n = 494) had at least 1 associated clinical symptom. Of these, 28.8% (204) of CNV-positive individuals had an NPD code in their electronic health record, compared with 13.3% (11 835 of 89 887) of CNV-negative individuals (odds ratio, 2.21; 95% CI, 1.86-2.61; *P* < .001); 66.4% (470) of CNV-positive individuals had a history of depression and anxiety compared with 54.6% (49 118 of 89 887) of CNV-negative individuals (odds ratio, 1.53; 95% CI, 1.31-1.80; *P* < .001). 16p13.11 (71 [0.078%]) and 22q11.2 (108 [0.119%]) were the most prevalent deletions and duplications, respectively. Only 5.8% of individuals (41 of 708) had a previously known genetic diagnosis. Results disclosure was completed for 141 individuals. Positive participant responses included poignant reactions to learning a medical reason for lifelong cognitive and psychiatric disabilities.

**Conclusions and Relevance:**

This study informs critical factors central to the development of population-based genomic screening programs and supports the inclusion of NPD in future designs to promote equitable access to clinically useful genomic information.

## Key Points

**Question:**

Are neuropsychiatric disorders that are associated with pathogenic copy number variants suitable for inclusion in population-based genomic screening programs?

**Findings:**

In this health care system population of more than 90 595 participants, copy number variants associated with neuropsychiatric disorders were prevalent and penetrant. Participant responses to receiving these genomic results were overall positive, suggesting personal utility.

**Meaning:**

Neuropsychiatric disorders associated with genetic variants should be considered in the design of population-based genomic screening programs.

## Teaser

This cohort study evaluates the suitability of including pathogenic copy number variants associated with neuropsychiatric disorders in population screening by determining their prevalence and penetrance and exploring the personal utility of disclosing results.

## Introduction

Genome-wide testing technologies, such as exome sequencing and chromosomal microarray, are being increasingly used across clinical settings as standard of care for identifying medically relevant genomic variants that cause a variety of conditions, including hereditary cancer syndromes, cardiovascular disorders, and neurodevelopmental disorders.^1,2,3,4,5,6,7,8,9,10^ Most testing still occurs within traditional clinical settings based on a patient’s personal or family history. However, comprehensive, population-based genomic screening programs, such as that carried out within the Geisinger MyCode Community Health Initiative, are quickly emerging as promising complementary public health options to proactively affect patient care.^11,12^ Several key factors have been described as critical to implement these programs, including results that inform disease prevention, early detection, or management; affect access to social services; and benefit family members.^13^ In addition, personal utility, equitable access to personally and medically relevant genomic information, and health care utilization effects are important factors.^13^ Furthermore, there is public interest for inclusion of genomic disorders that are often not designated as medically actionable, such as neuropsychiatric disorders (NPDs).^14,15^

Neuropsychiatric disorders, such as autism spectrum disorder, schizophrenia, and bipolar disorder, comprise an etiologically heterogeneous group of conditions affecting at least 14% to 18% of children and adults in the US.^16,17^ Lifetime prevalence for common psychiatric disorders, including depression and anxiety, has been estimated globally at 29%.^18^ Individuals with NPDs have higher than expected rates of chronic medical conditions and associated early mortality and account for a disproportionately large share of health care costs, including emergency department and inpatient visits and prescription drugs.^19,20^ Collectively, rare pathogenic copy number variants (CNVs) and single-gene sequence–level variants represent the largest proportion of known NPD causes to date, yet research into precision health strategies has lagged behind other conditions, such as cancer and cardiovascular disease.^7,21,22,23,24,25,26,27^

**Figure 1.**
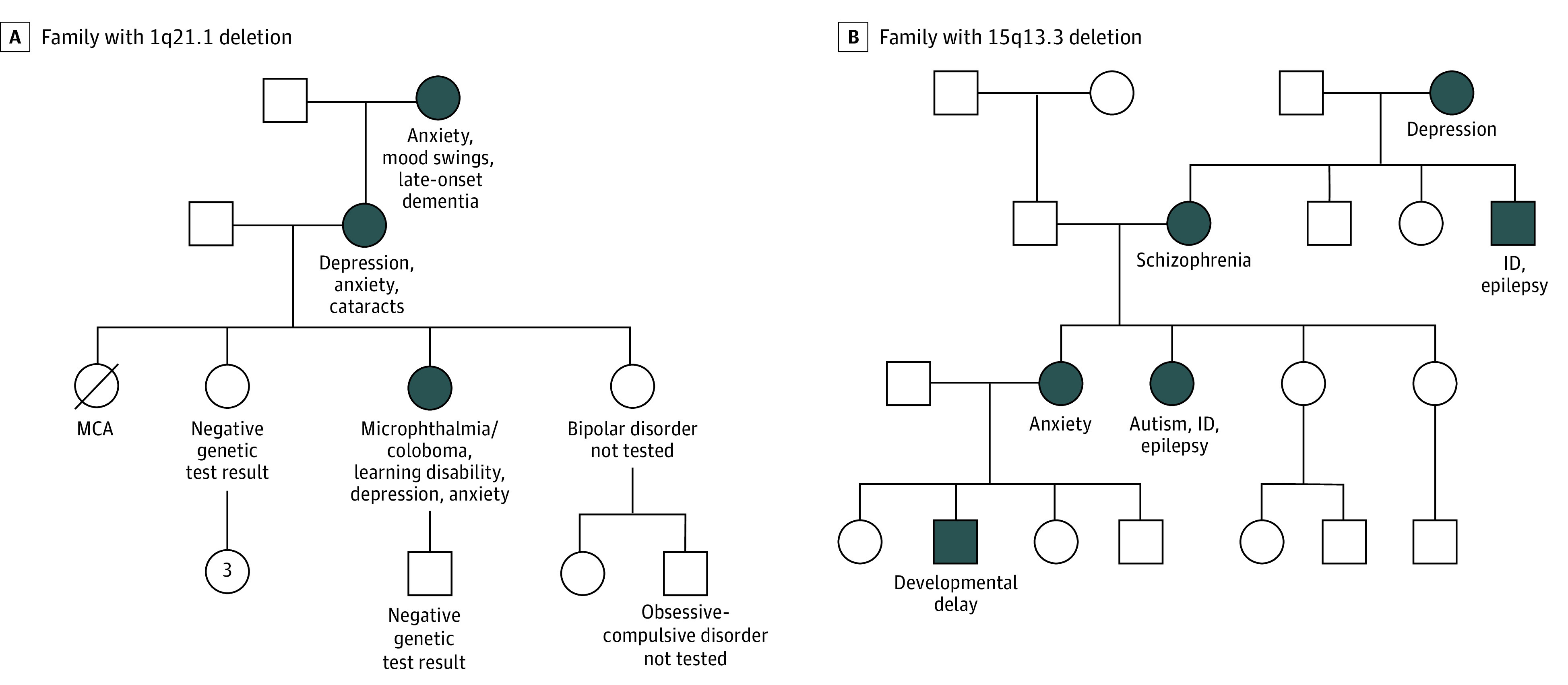
Pathogenic Copy Number Variants Exhibit Variable Expressivity Within Families Individuals designated in filled blue circles have tested positive for a copy number variant. Pedigrees are representative of family histories described by participants with pathogenic copy number variants. ID indicates intellectual disability; MCA, multiple congential anomalies.

Rare CNVs have large, primary effects on neuronal pathways and are causative of brain dysfunction, manifesting as clinically distinct disorders that exhibit a high degree of variable expressivity (Figure 1).^28^ Additionally, subclinical learning or social differences are often present, which may affect an individual’s well-being.^29^ Many NPD-associated genes and CNVs share biochemical and neurobiological pathways with other brain disorders, such as epilepsy and intellectual disability, that are frequent comorbidities in people with NPDs.^30,31^ The phenotypic variability of these large effects is modulated by family genomic background through the aggregate contribution of common variants of small effect size, quantified by polygenic risk scores,^32,33,34,35,36,37^ and may be further influenced by environmental experiences.

Genomic testing for NPDs and other brain disorders is increasingly embraced in the pediatric setting, with diagnostic yields for sequence variants and CNVs approaching 40%, and observations of NPD-associated CNVs in variably affected adults have informed our understanding of the clinical variability and natural history of these disorders.^1,7,28,29,38,39,40,41,42,43^ Hundreds of distinct genetic NPD causes are now known, representing a growing subset of pediatric- and adult-onset developmental and psychiatric diagnoses. However, clinical genetic testing in adult NPD populations is much less common, raising concerns about equitable access to highly relevant information for these individuals, and the penetrance of NPD-associated CNVs from an unselected population is less well known.

Our understanding of genomic contributors to human disease has gained momentum in recent years through analyses of large-scale research population data sets that provide a crucial counterbalance to clinically ascertained cohorts.^44,45,46^ Previous population-based studies have reported an estimated prevalence of approximately 1% for NPD-associated CNVs,^46,47,48^ indicating they may be as, or more, common than hereditary cancer and cardiovascular disorders currently included in population-based genomic screening programs. While these initiatives have allowed an unbiased assessment of population prevalence for genomic variants, they often lack detailed clinical correlation, particularly for NPD phenotypes. Furthermore, because these research initiatives do not have approval to return clinically confirmed and medically relevant information back to participants, there are very limited data on the perceived utility of this information from program participants.

To explore the suitability of including NPD-associated CNVs in population-based genomic screening programs, we assessed key factors incorporated into development and implementation decision-making frameworks: prevalence, penetrance, and early indications of participant response and personal utility. We report evidence from the first study, to our knowledge, of a large-scale, health care system–based population, using a linked electronic health record (EHR) and genomic data set, that supports inclusion of NPD-associated CNVs in population-based genomic screening and provides experience on the feasibility and logistical components for implementation.

## Methods

The cohort for this study was identified from Geisinger’s MyCode Community Health Initiative, a biorepository with more than 250 000 consented health care system patient-participants, unselected for age or sex and with approximately 97.7% European ancestry.^49^ We evaluated the frequency of 31 pathogenic, recurrent CNVs (eTable 1 in the Supplement) from exome sequencing data available from the DiscovEHR cohort (n = 92 455), a subset of MyCode participants with paired exome and EHR data. All DiscovEHR participants provided written consent to be contacted through our Genomic Screening and Counseling program about clinically relevant genomic results. The institutional review board at Geisinger approved this study. The DiscovEHR cohort was recruited from February 2007 to April 2017. Data were collected for the qualitative analysis April 2017 through February 2018. The disclosures were conducted March 2017 through March 2019. The most recent electronic health records data were extracted in January 2019.

DNA sample preparation and exome sequencing were performed in collaboration with the Regeneron Genetics Center as previously described.^50,51^ CNV calling was carried out on exome data using the copy number estimation using the lattice-aligned mixture models algorithm.^52^ After quality control filtering, data from 90 595 participants were included for CNV analysis. CNVs with a ClinGen Dosage Sensitivity score of 3 were selected for evaluation.^53^ Participants were designated as CNV-positive if break points overlapped at least 50% of the defined region (eTable 1 in the Supplement).^48^ Most calls (688 [96%]) spanned at least 90% of the defined region. CNVs called from exome data were confirmed by an array-based method using PennCNV or manual inspection of the signal intensity data.^54^

To assess the frequency of relevant CNV-related phenotypes in CNV-positive individuals compared with CNV-negative individuals, *International Classification of Diseases, Ninth Revision* and *International Statistical Classification of Diseases and Related Health Problems, Tenth Revision* billing codes (eTable 2 in the Supplement) were extracted from linked EHRs. Nine pathogenic CNVs were prioritized for participant disclosure, following established protocols,^12^ after confirmation in a clinical laboratory (100% confirmed). A mixed-method approach was used to evaluate participants’ responses to receiving results, with written consent provided for audio recording of in-person disclosure sessions. Age was calculated based on the last encounter in EHR. Tests of association were performed with logistic regression adjusted for age and sex. Reported *P* values were 2-sided, and statistical significance was set at .005. Reported *P *values are Bonferroni corrected for the 9 tests of association performed in the study. All statistical analyses were performed using R statistical software version 3.4.1 (R Project for Statistical Computing). Analysis began February 2018 and ended December 2019. A detailed description is provided in the eMethods in the Supplement.

## Results

### Prevalence of NPD-Associated CNVs

Of the 90 595 DiscovEHR patient-participants for whom exome sequencing and CNV calling passed quality control (eMethods in the Supplement), 708 (0.8%) had 1 of the 31 CNVs of interest (Table 1); 436 (61.6%) were female and 272 (38.4%) were male, consistent with the general DiscovEHR cohort. Duplications of 22q11.2 (108 [0.119%]) were the most prevalent CNV overall, followed by 1q21.1 duplications (90 [0.099%]). The most prevalent deletion was 16p13.11 (71 [0.078%]). Duplications and deletions of 16p11.2 (63 [0.070%] and 59 [0.065%], respectively) were the next most prevalent CNVs. The cumulative prevalence (708 [0.8%]) of individuals with a pathogenic CNV is comparable with reports by other population-based studies, such as the UK Biobank (1%),^48^ Estonian Genome Center of the University of Tartu (0.7%),^46^ and deCODE (1.16%).^47^ However, when a direct comparison was done across cohorts limiting the analysis to our 31 CNVs of interest, the DiscovEHR prevalence rates were notably higher for some CNVs (eg, 15q13.3), possibly due to ascertainment bias or technical differences with previous studies (Table 1).

**Table 1.** Comparison of NPD-Associated CNV Prevalence in DiscovEHR, deCODE, EGCUT, and UK Biobank

CNV	Dosage	No. (%)			
		DiscovEHR (n = 90 595)	deCODE^47^ (n = 101 655)	EGCUT^46^ (n = 7877)	UK Biobank^48^ (n = 421 268)
Deletions					
1q21.1 (*GJA5*)^a^^,^^b^	del	59 (0.065)	35 (0.034)	3 (0.038)	113 (0.027)
3q29 (*DLG1*)	del	4 (0.004)	3 (0.003)	0	9 (0.002)
5q35 (*NSD1*)	del	0	NR	0	0
7q11.23 (*ELN*)^a^	del	4 (0.004)	4 (0.004)	1 (0.013)	1 (0)
8p23.1 (*GATA4*)	del	0	NR	1 (0.013)	4 (0.001)
10q23 (*BMPR1A*)	del	1 (0.001)	NR	NR	3 (0.001)
15q11.2q13.1 BP1-3 (*UBE3A*)	del	5 (0.006)	1 (0.001)	0	1 (0)
15q13.3 BP4-5 (*CHRNA7*)^a^	del	55 (0.061)	25 (0.025)	2 (0.025)	42 (0.010)
15q24 (*SIN3A*)^a^	del	2 (0.002)	NR	0	1 (0)
16p13.11 (*MYH11*)^a^	del	71 (0.078)	38 (0.037)	2 (0.025)	131 (0.031)
16p11.2 distal (*SH2B1*)	del	28 (0.031)	19 (0.019)	NR	58 (0.014)
16p11.2 (*TBX6*)^a^	del	59 (0.065)	43 (0.042)	4 (0.051)	110 (0.026)
17p12 (*PMP22*)	del	31 (0.034)	32 (0.031)	3 (0.038)	237 (0.056)
17p11.2 (*RAI1*)	del	4 (0.004)	NR	0	2 (0)
17q11.2 (*NF1*)^a^	del	3 (0.003)	NR	0	9 (0.002)
17q12 (*HNF1B*)^a^	del	4 (0.004)	7 (0.007)	0	9 (0.002)
17q21.31 (*KANSL1*)	del	0	NR	0	0
22q11.2 (*TBX1*)^a^^,^^c^	del	23 (0.025)	18 (0.018)	0	10 (0.002)
22q11.2 distal	del	1 (0.001)	1 (0.001)	0	5 (0.001)
Duplications					
1q21.1 (*GJA5*)^b^	dup	90 (0.099)	60 (0.059)	6 (0.076)	177 (0.042)
5q35 (*NSD1*)	dup	0	NR	0	0
7q11.23 (*ELN*)	dup	8 (0.009)	1 (0.001)	1 (0.013)	14 (0.003)
8p23.1 (*GATA4*)	dup	0	NR	0	6 (0.001)
15q11.2q13.1 BP1-3 (*UBE3A*)	dup	3 (0.003)	13 (0.013)	0	19 (0.005)
16p11.2 (*TBX6*)	dup	63 (0.07)	51 (0.050)	7 (0.089)	138 (0.033)
17p12 (*PMP22*)	dup	38 (0.042)	28 (0.028)	2 (0.025)	124 (0.029)
17p11.2 (*RAI1*)	dup	0	NR	0	5 (0.001)
17q11.2 (*NF1*)	dup	4 (0.004)	NR	NR	2 (0)
17q12 (*HNF1B*)	dup	41 (0.045)	38 (0.037)	7 (0.089)	101 (0.024)
22q11.2 (*TBX1*)^d^	dup	108 (0.119)	85 (0.084)	5 (0.063)	280 (0.066)
22q11.2 distal	dup	1 (0.001)	NR	0	13 (0.003)
Deletion	NA	354 (0.391)	226 (0.222)	16 (0.203)	745 (0.177)
Duplication	NA	356 (0.393)	276 (0.272)	28 (0.355)	879 (0.209)
CNV^e^	NA	708 (0.782)	502 (0.494)	44 (0.559)	1624 (0.386)

Abbreviations: CNV, copy number variant; del, deletion; dup, duplication; EGCUT, Estonian Genome Center of the University of Tartu; NPD, neuropsychiatric disorder; NA, not applicable; NR, not reported.

^a^ Deletions targeted for disclosure to patient-participants.

^b^ One 1q21.1 deletion and seven 1q21.1 duplications also include the TAR region.

^c^ Of 22q11.2 deletions, 8 and 15 overlapped with the A-B and A-D coordinates, respectively

^d^ Of 22q11.2 duplications, 19 and 89 overlapped with the A-B and A-D coordinates, respectively.

^e^ In calculating the prevalence of individuals with CNVs, we account for 2 individuals who each had 2 CNVs in the DiscovEHR cohort. In deCODE, EGCUT, and UK Biobank, cumulative CNV prevalence in the study population is estimated based on the sum of the individual CNV frequencies and may represent a slight overestimate.

### Estimated Penetrance of NPD-Associated CNVs

We estimated the penetrance of NPD and congenital malformations in individuals with CNVs. We found that 28.8% (204 of 708) of CNV-positive individuals had an NPD-associated code in their EHR (eTable 3 in the Supplement) compared with 13.3% (11 835 of 89 887) of CNV-negative individuals (odds ratio [OR], 2.21; 95% CI, 1.86-2.61; *P* < .001). When we broadened our NPD definition to include 2 common psychiatric disorders, depression and anxiety, we observed 66.4% (470 of 708) of CNV-positive individuals with this history compared with 54.6% (49 118 of 89 887) of CNV-negative individuals (OR, 1.53; 95% CI, 1.31-1.80; *P* < .001).

Congenital malformations in 1 of 5 categories (central nervous system, cardiac, kidney/urinary, genital, cleft lip/palate) were observed in 13.3% (94 of 708) of CNV-positive individuals (eTables 4 and 5 in the Supplement), compared with 7.4% (6683 of 89 887) of CNV-negative individuals (OR, 2.00; 95% CI, 1.60-2.49; *P* < .001). Cardiac defects were the most common congenital malformations observed in 6.5% (46 of 708) of CNV-positive individuals followed by congenital malformations affecting the urinary system in 2.9% (21 of 708), central nervous system in 2.3% (16 of 708), and genital organs in 2.1% (15 of 708). Cleft lip and/or palate were observed in 0.99% (7 of 708) of CNV-positive individuals, a 9.84 higher odds (95% CI, 4.07-20.24; *P* < .001) than CNV-negative individuals (0.08% [68 of 89 887]). The prevalence estimates of other congenital malformations were approximately 2- to 3-fold higher in the CNV-positive group when compared with CNV-negative individuals, including cardiac (2.9% [2647 of 89 887]; OR, 2.48; 95% CI, 1.81-3.32; *P* < .001), central nervous system (0.83% [750 of 89 887]; OR, 2.20; 95% CI, 1.28-3.51; *P* = .02), and genital malformations (0.9% [824 of 89 887]; OR, 2.00; 95% CI, 1.14-3.23; *P* = .08). In contrast, congenital malformations affecting the urinary system were observed at an equal prevalence of 3.0% (21 of 708; OR, 1.15; 95% CI, 0.72-1.73; *P* = .53), in CNV-positive (21 of 708) and CNV-negative (2716 of 89 887) individuals. Overall, 69.8% (494 of 708) of CNV-positive individuals had NPD, including depression and anxiety, or a congenital malformation compared with 57.5% (51 645 of 89 887) of CNV-negative individuals (OR, 1.61; 95% CI, 1.37-1.90; *P* < .001).

Despite this high rate of documented CNV-related clinical symptoms, only a minority of individuals (5.8% [41 of 708]) had an EHR-documented genetic diagnosis. The mean (SD) age of participants with a genetic diagnosis was 20.33 (14.53) years (compared with 50.04 [18.74] years for all CNV-positive individuals), reflecting a strong bias toward offering clinical genetic testing to younger individuals. For the subset of individuals with 1 of 9 CNVs prioritized for disclosure, 77.1% (216 of 280) had a relevant NPD or congenital malformation code (49.6% [139 of 280] after excluding depression and/or anxiety) and only 8.6% (24 of 280) had a known genetic diagnosis.

### Participant Experience and Personal Utility of NPD-Associated Results

**Figure 2.**
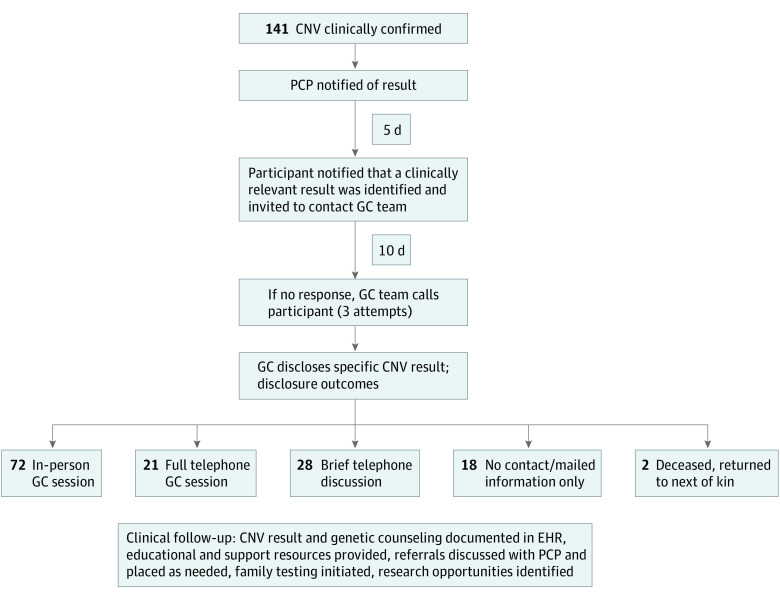
Genetic Screening and Counseling Disclosure Process and Outcomes From 141 CNV-Positive Participants CNV indicates copy number variant; EHR, electronic health record; GC, genetic counselor; PCP, primary care professional.

The results disclosure process was completed for 141 DiscovEHR patient-participants with pathogenic NPD-associated CNVs. Table 2 shows the number of participants identified with each of the 9 prioritized CNVs, as well as the sex distribution, mean age, and NPD diagnoses in the EHR. One hundred twenty-one individuals (85.8%) actively responded to invitations to receive genetic counseling and learned of their diagnoses through in-person appointments and/or telephone sessions, if requested owing to transportation limitations. Of these, 72 (59.5%) had in-person counseling, 21 (17.3%) participated in full telephone counseling sessions, and 28 (23.1%) had initial telephone discussions about the diagnosis but declined further counseling or could not be recontacted for scheduling. These participants were mailed a detailed summary letter and packet of information about their CNV diagnoses, with genetic counselor contact information. Participants’ primary care professionals were notified of all results-related communications, and genetic counselors partnered with clinicians to direct medical care (Figure 2).

**Table 2.** NPD Phenotypes Among 141 Individuals With Disclosed CNVs

CNV	Total participants	Male	Female	Mean (SD) age at disclosure, y	NPD diagnoses in EHR
1q21.1 deletion	28	5	23	53.96 (17.35)	Depressive disorder (n = 21), anxiety disorder (n = 19), adjustment disorder (n = 12), bipolar/mood disorder (n = 3), psychosis (n = 2), ADHD (n = 1)
7q11.23 deletion	2	0	2	50.50 (21.92)	Depressive disorder (n = 1), anxiety disorder (n = 1), bipolar/mood disorder (n = 1), schizophrenia (n = 1)
15q13.3 deletion	29	9	20	46.93 (13.69)	Depressive disorder (n = 18), anxiety disorder (n = 15), adjustment disorder (n = 8), seizure disorder (n = 6), mood disorder (n = 5), learning disorder/intellectual disability (n = 4), obsessive compulsive disorder (n = 2), psychosis (n = 2), ADHD (n = 1)
15q24 deletion	0	0	0	NA	NA
16p11.2 deletion	29	13	16	48.66 (12.54)	Depressive disorder (n = 19), anxiety disorder (n = 15), adjustment disorder (n = 10), intellectual disability (n = 5), seizure disorder (n = 4), eating disorder (n = 2), ADHD (n = 1), mood disorder (n = 1), obsessive compulsive disorder (n = 1), Tourette syndrome (n = 1)
16p13.11 deletion	39	13	26	52.51 (15.32)	Depressive disorder (n = 26), anxiety disorder (n = 26), adjustment disorder (n = 12), seizure disorder (n = 11), bipolar/mood disorder (n = 5), developmental delay/intellectual disability (n = 4), psychosis (n = 1), ADHD (n = 1), Tic disorder (n = 1)
17q11.2 deletion	3	0	3	48.67 (7.57)	Depressive disorder (n = 3), anxiety disorder (n = 1), adjustment disorder (n = 1), ADHD (n = 1)
17q12 deletion	4	2	2	43.75 (25.01)	Adjustment disorder (n = 3), depressive disorder (n = 2), anxiety disorder (n = 2), ADHD (n = 1), autism spectrum disorder (n = 1), bipolar/mood disorder (n = 1), intellectual disability (n = 1), seizure disorder (n = 1)
22q11.2 deletion	7	4	3	36.29 (13.06)	Developmental delay/intellectual disability (n = 4), depressive disorder (n = 3), anxiety disorder (n = 3), bipolar disorder/mood disorder (n = 3), adjustment disorder (n = 2), schizophrenia (n = 2), seizure disorder (n = 2), psychosis (n = 1), language disorder (n = 1), autism spectrum disorder (n = 1), ADHD (n = 1)
Total	141	46	95	49.70 (15.28)	NA

Abbreviations: ADHD, attention-deficit/hyperactivity disorder; CNV, copy number variant; EHR, electronic health record; NA, not applicable; NPD, neuropsychiatric disorder.

Participant perspectives on receiving CNV diagnoses were collected via genetic counselor postsession assessments (38 [27%]; eFigure in the Supplement) and audio recordings (14 [10%]). Qualitative analysis of these 2 data sets revealed positive major themes demonstrating the value of reporting these results to participants (Table 3). Analysis of a larger data set of audio-recorded session transcripts will be reported separately.

**Table 3.** Thematic Analysis of Participant Responses to Receiving NPD-Associated CNVs

Major themes	Exemplary quotes
Discussed NPD history that was not recorded in EHR	“I was a slow learner.” (Female, 17q11.2)
	“I was left out… I was different from other kids.” (Female, 1q21.1)
Previously had explained NPD as caused by social circumstances (n = 17 from GC notes; n = 6 from transcripts)	“I do put a lot of [my learning disability on] what happened between Mom and Dad and the moving around.” (Male, 16p11.2)
Expressed that the CNV fit or made sense with lived experience (n = 22 from GC notes; n = 10 from transcripts)^a^	“I knew I had anxiety. I knew I had different things, but I didn’t know where everything came from. This now brings everything around.” (Female, 1q21.1)
Felt reassured that NPD was not their fault (n = 9 from GC notes; n = 2 from transcripts)^a^	“It was very helpful. It took a lot of guilt off.” (Mother of Male, 22q11.2)
Reported that sense of self stayed the same or improved (n = 36 from GC notes; n = 14 from transcripts)	“I think it does [change sense of self], because I realize there’s a medical…, that’s something behind everything. It’s not just all in your head.” (Female, 1q21.1)
Positive and negative emotions were expressed together (n = 7 from transcripts)^b^	“I thought it was something bad, but it’s bad and a good thing at the same time, that information that you gave me.” (Female, 17q11.2)
Information was valuable for themselves and family members (n = 32 from GC notes; n = 14 from transcripts)^a^	“It feels good to know that there’s a name for my condition.” (Male, 22q11.2)
	“If this information is something that we can help [our son]… We can get a little bit more control of it now.” (Wife of male, 16p13.11)
Showed resilience through life experience and for the future (n = 9 from transcripts)^b^	“I’ll just learn to deal with it [CNV result].” (Female, 16p11.2)

Abbreviations: CNV, copy number variant; EHR, electronic health record; GC, genetic counselor; NPD, neuropsychiatric disorders.

^a^ Theme directly related to personal utility.

^b^ Not coded in GC notes.

Participant responses were overall positive or neutral (36 of 38 [95%] from genetic counselor assessments; 13 of 14 [93%] from audio recording transcripts). Many participants revealed learning issues, social difficulties, or hospitalizations for mental health issues that were not captured in the EHR. It was common for participants to have a perceived explanation for their personal and/or family history of NPD, often attributing it to social circumstances (eg, trauma, family disruption, history of abuse). Participants expressed relief and satisfaction at finally having a medical explanation for a lifelong history of learning and behavioral struggles. The CNV result reassured them that their NPD history was not their fault, and several expressed that they wished they had known this information earlier in life. Most participants (22 of 38 [58%] from genetic counselor assessments; 10 of 14 [71%] from transcripts) observed that their genetic diagnosis fit or made sense with their personal or medical histories.

Participants typically stated that they planned to share their genetic test results with family members and often brought a support person to the disclosure session. Concerns about the potential for NPD in their children and grandchildren were a motivation for communicating results within a family, especially given the 50% chance of inheriting the CNV. However, a subset (6 of 38 [16%] from genetic counselor assessments; 4 of 14 [29%] from transcripts) indicated that they may not share the information broadly with family members who are less supportive of them. To date, cascade testing has been completed for 26 family members resulting in 16 newly detected CNV diagnoses, primarily in participants’ adolescent and young adult offspring, 94% of whom had clinically diagnosed NPD of previously unknown origin.

## Discussion

Population-based genomic screening programs are a promising element to the cost-effective implementation of genomic medicine.^13,55^ These programs have the potential to have broad reach and ensure individuals with medically relevant genomic variants are identified proactively, avoiding the selection bias inherent in current clinical referral-based systems, which fail to identify a significant proportion (reported estimates up to 50%) of at-risk individuals.^56,57,58^ In addition to the value that population screening can have on disease management, several other concepts have been recognized as critical outcomes, including access to social services, personal utility, health care utilization optimization, equal access, and communication of results within health care systems and families.^13^

This study illustrates that NPD-associated CNVs are both prevalent and penetrant in a health care system–based population. Nearly 1% of MyCode participants have an NPD-associated CNV, collectively exceeding the prevalence estimates of disorders regularly included in population-based screening programs, such as familial hypercholesterolemia (1 in 200 to 250 [0.4%-0.5%]),^59^ Lynch syndrome (1 in 440 [0.2%]),^60^ or hypertrophic cardiomyopathies (1 in 500 [0.2%]).^61^ This population CNV prevalence is consistent with previous estimates from European populations,^46,47,48^ and additional study of more diverse populations is warranted. Differences between our data and previous estimates, most notably for 15q13.3 deletion, could be explained by ascertainment bias because individuals with more severe NPD may be more likely to be included in our population.^62^ Furthermore, because our CNV detection used both exome and microarray methods, with secondary clinical confirmation, our true positive rate could be increased. Research exploring the inclusion of NPD-associated single-gene disorders is also recommended to more completely represent all genetic causes of NPD.

The medical histories of MyCode participants with NPD-associated CNVs reflect the elevated risk for NPD and congenital malformation. Approximately 35% of all CNV-positive individuals, and 77% of those with a pathogenic CNV prioritized for participant disclosure, had documented NPD or congenital malformation in their medical records. Additionally, our data are consistent with previous observations that individuals with rare CNVs are at increased risk for depression.^63^ These penetrance estimates (35%-70%) are comparable with some of the highest health risks associated with hereditary cancer and cardiovascular disorders, including *BRCA1/2* (38%-87% lifetime risk for breast cancer), Lynch syndrome (52%-82% lifetime risk for colorectal cancers), and familial hypercholesterolemia (30%-50% risk for coronary event).^59,60,64^ Genetic causes will increasingly inform NPD care by hastening diagnosis and treatment of developmental or psychiatric concerns (eg, schizophrenia with 22q11.2 deletions) and by monitoring for known medical risks (eg, kidney disease and maturity onset diabetes of the young with 17q12 deletions).^65^

There is growing recognition of the broader benefits to patients learning genomic results, beyond the conservative and cost-centric requirement of medical actionability, including consideration of the personal utility of genomic information, which requires that genomic information be “used for decisions, actions, or self-understanding which are personal in nature.”^13,66,67^ A genomic result in a given patient could have relatively few medical management implications but be perceived as highly valuable in personal or social ways.^14,15,68,69,70^ These benefits can influence decisions to adhere to medical recommendations, one’s ability to seek or obtain social or educational support services, family communication about NPD, and self-understanding.^68,69,70,71,72,73,74,75^ Participants in this study expressed several indications of personal utility, including the value of having a medical explanation for their disabilities. This notion was often expressed as a sense of reassurance that they are not at fault for their NPD, a validation that their learning and/or psychiatric difficulties were real, and an indication of enhanced understanding of themselves and their families. The personal utility of finding an etiological diagnosis may prove to be among the most important benefits of genomic testing for NPD.

Clinically defined NPD, such as autism spectrum disorder, schizophrenia, and bipolar disorder, are intertwined at a biological level by shared genomic underpinnings that can be readily diagnosed through clinically available genetic testing.^28^ However, only 5.8% of individuals in our cohort had previously received a genetic diagnosis, demonstrating that the care received by most adults with NPD-associated CNVs has not been informed by a genetic cause. Instead, most individuals live with symptom-based developmental, psychiatric, and medical diagnoses without knowing the underlying genetic cause that ties these findings together. The majority of study participants were already adults when submicroscopic CNVs were first discovered, and, because clinical testing for NPD-associated CNVs is not often offered to adults outside of parental testing in a pediatric setting, were unlikely to have had access to diagnostic testing that is now considered standard of care for pediatric developmental disorders and congenital malformations. Thus, adults with NPD are unlikely to have access to genomic information of high clinical and personal relevance to them and their family members. Importantly, 94% of participants’ family members who were identified as having a CNV by cascade testing had clinically diagnosed NPD of unknown cause, further highlighting the unmet need for genomic testing in this population.

Public views about receiving NPD-associated genomic results, as well as genomic results associated with nonactionable diseases, generally lean toward ensuring access and disclosure.^14,15,76^ The medical community, including psychiatry, is also beginning to respond to these public views, moving away from a historically paternalistic approach to genomic result disclosure.^15,77^ Psychosocial outcomes data also indicate that patient-participants adjust to genomic information with little long-term negative consequences.^57,71,78^ Identifying genetic diagnoses for individuals with NPD allows us to move beyond vague discussions about multifactorial risk to more targeted, medical explanations.

Ongoing research is needed to further document clinical, psychological, and family outcomes of CNV results disclosure and to inform development of disease-specific clinical models and support tools to complement population-based genomic screening. Additionally, research that incorporates polygenic risk and other factors could inform CNV-specific risk estimates for particular NPD and congenital malformation diagnoses. We anticipate that medicalizing NPD through the identification of specific genetic causes, paired with targeted genetic counseling and family cascade testing, may decrease stigma, increase self-advocacy, and lead to closer engagement of NPD patients with health care clinicians. These improvements could ultimately translate into more cost-effective utilization of health care resources and improved compliance with treatment recommendations.

### Limitations

Our prevalence and penetrance estimates of pathogenic CNVs in DiscovEHR likely underestimate the true prevalence in the general population and represent a milder range of observed clinical phenotypes. While we did not exclude individuals with more severe cognitive and psychiatric disabilities from this study, they are likely underascertained in the DiscovEHR cohort owing to difficulty ensuring informed research consent or use of specialized medical care outside the general health care system, such as residential psychiatric facilities. Furthermore, our penetrance estimates relied on structured EHR data; many participants shared additional NPD histories during disclosure sessions that were not formally documented in the EHR. The advanced age of our participants also likely introduced a survival bias due to underrepresentation of individuals with severe medical conditions, such as CNV-related congenital cardiac anomalies, that reduce life expectancy.

## Conclusions

This study of a large, health care system population demonstrates that there is a significant proportion of our population that could benefit from including NPD in population-based genomic screening programs. We have established that NPD-associated CNVs have sufficient prevalence and penetrance to be considered in the development and clinical implementation of such programs. Furthermore, we conclude that identification and disclosure of causative genomic variants is clinically and personally valuable for individuals with NPD and their families, who are likely to be underserved and have more limited access to this meaningful information.^79^

## Notes

Supplement. eMethods. eReferences. eTable 1. Genomic Coordinates and Inclusion Criteria for Copy Number Variants eTable 2. ICD-9 and ICD-10 Codes for Neuropsychiatric Disorders, Congenital Malformations, and Chromosomal Abnormalities eTable 3. Neuropsychiatric Disorder Diagnoses by Copy Number Variant (Excluding Depression and Anxiety) eTable 4. Congenital Malformation Diagnoses by Copy Number Variant eTable 5. Combined Congenital Malformation and Neuropsychiatric Diagnoses by Copy Number Variant eFigure. Genetic Counselor Results Disclosure Session Outline
