# Supplementary material for: Identification of Neuropsychiatric Copy Number Variants in a Health Care System Population
Source: JAMA Psychiatry. 2020 Jul 22;77(12):1–10. doi: 10.1001/jamapsychiatry.2020.2159 (PMC7376464; doi:10.1001/jamapsychiatry.2020.2159)
Supplement: Supplement. — eMethods. eReferences. eTable 1. Genomic Coordinates and Inclusion Criteria for Copy Number Variants eTable 2. ICD-9 and ICD-10 Codes for Neuropsychiatric Disorders, Congenital Malformations, and Chromosomal Abnormalities eTable 3. Neuropsychiatric Disorder Diagnoses by Copy Number Variant (Excluding Depression and Anxiety) eTable 4. Congenital Malformation Diagnoses by Copy Number Variant eTable 5. Combined Congenital Malformation and Neuropsychiatric Diagnoses by Copy Number Variant eFigure. Genetic Counselor Results Disclosure Session Outline [file jamapsychiatry-e202159-s001.pdf]

Supplementary Online Content

Martin CL, Wain KE, Oetjens MT, et al. Identification of neuropsychiatric copy number variants in a health care system population. *JAMA Psychiatry*. Published online July 22, 2020. doi:10.1001/jamapsychiatry.2020.2159

eMethods.

eReferences.

eTable 1. Genomic Coordinates and Inclusion Criteria for Copy Number Variants

eTable 2. ICD-9 and ICD-10 Codes for Neuropsychiatric Disorders, Congenital Malformations, and Chromosomal Abnormalities

eTable 3. Neuropsychiatric Disorder Diagnoses by Copy Number Variant (Excluding Depression and Anxiety)

eTable 4. Congenital Malformation Diagnoses by Copy Number Variant

eTable 5. Combined Congenital Malformation and Neuropsychiatric Diagnoses by Copy Number Variant

eFigure. Genetic Counselor Results Disclosure Session Outline

This supplementary material has been provided by the authors to give readers additional information about their work.

© 2020 Martin CL et al. *JAMA Psychiatry.*

eMethods.

DiscovEHR Cohort

The cohort for this study was identified from Geisinger's MyCode® Community Health Initiative, a biorepository operating in a CLIA-laboratory environment with >250,000 consented patient-participants, unselected for age or gender, receiving medical care at Geisinger[1]. Exome sequencing (ES) and linked EHR data are currently available for 92,455 MyCode participants - a subset known as the DiscovEHR cohort – on which this study is based. The DiscovEHR cohort is highly homogeneous in terms of European ancestry (97.7%), based on participant report, and primarily includes older adults (mean age: 57 years) recruited from an unselected Geisinger outpatient population with a median follow-up of 14.6 years of EHR-linked data. More than half of the participants are female (60.6%). All DiscovEHR participants provided written consent (IRB #2006-0258) to be contacted through our Genomic Screening and Counseling (GSC) program about clinically relevant genomics results. The list of reportable gene-disease pairs is dynamic and can be updated to add or remove genes or genomic regions as evidence is acquired. It currently is comprised of 61 gene-disease pairs, including those on the American College of Medical Genetics and Genomics (ACMG) secondary findings list[2].

Exome Sequencing, SNP Genotyping, and CNV Calling

DNA sample preparation and ES for 92,455 DiscovEHR participants were performed in collaboration with the Regeneron Genetics Center, as described previously[3,4]. In brief, VCRome exome capture probes (Nimblegen, Madison, Wisconsin) were used for the first ~61K samples sequenced and a modified version of xGen probes (Integrated DNA Technologies, Coralville, Iowa) was used for the remaining ~31K samples. Samples were sequenced using 75 basepair paired-end sequencing on a v4 HiSeq 2500 (Illumina, San Diego, CA) to a coverage depth sufficient to provide greater than 20X haploid read depth of over 85% of targeted bases in 96% of samples (approximately 80X mean haploid read depth of targeted bases). Sequence reads were aligned to human reference GRCh38 with BWA-mem[5] and the resultant BAM files were processed using Picard (http://broadinstitute.github.io/picard) MarkDuplicates tool to identify and flag duplicate reads for exclusion in downstream analyses. CNV calling was carried out on ES data from 92,387 patient-participants using the Copy number estimation using Lattice-Aligned Mixture ModelS (CLAMMS) algorithm[6]. As part of the CLAMMS QC pipeline, samples with >40 CNV calls or full or partial aneuploidies (n=1,792) were excluded and our analysis was performed on the remaining 90,595 patient-participants.

CNV Selection and Validation

We evaluated the frequency of 31 pathogenic, recurrent, segmental duplication-mediated CNVs in the DiscovEHR cohort. CNVs were selected if there was strong evidence for pathogenicity documented by a Dosage Sensitivity Score of 3, as defined by the Clinical Genome Resource (ClinGen) Dosage Sensitivity Map (https://www.ncbi.nlm.nih.gov/projects/dbvar/clingen/), which uses an evidence-based process to evaluate CNV regions for clinically relevant haploinsufficiency and triplosensitivity[7]. The 31 selected CNVs are all well-established, pathogenic CNVs commonly reported as such by clinical genetic laboratories and known to cause NPD and, often, other medical concerns. Patient-participants were designated as positive for a CNV of interest if breakpoints overlapped at least 50% of the defined region (eTable 1)[8]. Most of the calls (n=688, 96%) spanned at least 90% of the defined region. Nearly all sequenced participants (>97%) were also genotyped on either the HumanOmniExpress (HOEE, Illumina; n=59,499) or Global Screening Array (GSA, Illumina; n=31,062) platforms. CNVs called from ES data were confirmed by an array-based call using PennCNV or manual inspection of the signal intensity data (logR and B-allele frequency) within the pathogenic region[9]. ES-called CNVs without array data were included if confirmed by a clinical laboratory as a part of this project or a diagnosis of the genetic disorder was present in the EHR (described below).

Clinical Data Extraction

The frequency of clinically related phenotypes among individuals with NPD-related CNVs was assessed by extracting relevant ICD-9/10 billing codes from the EHR (eTable 2). Our approach broadly captures developmental and psychiatric disorders of varying levels of severity that can be caused by a NPD-related CNV. The neuropsychiatric disorder ICD codes were chosen to capture disorders classified in the DSM-5 as "neurodevelopmental disorders," "schizophrenia spectrum and other psychotic disorders," "bipolar and related disorders," "depressive disorders," "anxiety disorders," and "obsessive-compulsive and related disorders" plus epilepsy and cerebral palsy, which are not included in the DSM-5 classification system. Congenital malformation (CM) categories (e.g., cardiac anomalies, cleft lip) were included as relevant phenotypes in our assessment of the

burden of disease that may be caused by NPD CNVs. Five CM categories were selected based on relevance to known CNV-related phenotypes and highest confidence in the accuracy of EHR-derived ICD-9/10 codes (eTable 2). A genetic diagnosis of a NPD-related CNV in the EHR was identified by the presence of an ICD-9/10 billing code indicating a chromosomal/structural abnormality (eTable 2). Tests of association were performed with logistic regression adjusted for age and sex. Thirteen individuals, missing sex information in their EHR were excluded from these analyses. Reported p-values are Bonferroni corrected for the nine tests of association performed in the study. All statistical analyses were performed using R version 3.4.1. Penetrance was calculated using the formula below:

$$\text{CNV penetrance} = \frac{\text{Number of cases with CNV}}{\text{Number of controls with CNV}}$$

Disclosure Protocol and Assessment of Participant Responses

A subset of nine pathogenic CNVs were prioritized to be returned to participants, based on the frequency in our dataset and the likelihood that the CNV could also cause non-NPD health considerations (e.g., congenital cardiac anomaly) that would be more likely to have implications for medical care. ICD9/10 codes were manually reviewed for individuals with a prioritized CNV to assess for evidence of an NPD phenotype. Individuals with one of these CNVs and a documented NPD, including depression and anxiety, in their EHR were eligible for clinical disclosure of results. DNA from samples maintained in the MyCode® biorepository CLIA environment were sent for orthogonal confirmation in a CAP- and CLIA-certified clinical laboratory and 141 (100%) results were confirmed. The clinical confirmation test report was scanned into the EHR and the disclosure protocol initiated.

We developed a results disclosure process to return prioritized NPD-related CNVs to adult patient-participants (Figure 2), based on the existing MyCode® GSC program[10]. The proposed CNV disclosure process and returnable CNVs underwent review by Geisinger's Ethics Advisory Council, Clinical Oversight Committee, Genomics Council, and MyCode® Governing Board prior to implementation.

Three licensed certified genetic counselors experienced with NPD were responsible for contacting and counseling participants about results, facilitating cascade testing for relevant family members, and providing clinical guidance to other healthcare providers. Initiation of result notification occurred from March 2017 to March 2019. A disclosure session outline (eFigure 1) was developed to ensure consistency among genetic counselors and to guide psychosocial assessment of participants' reactions. Particular consideration was given to reactions upon learning about a genetic NPD etiology and the effects of cognitive and psychiatric symptoms on the comprehension of information provided. Discussion included a description of the CNV, inheritance, knowledge of the associated clinical manifestations, the variable expressivity inherent to brain disorders, and the complex interplay between high-impact genomic variants and environmental factors. Personal medical and NPD histories and family histories were elicited, including participants' experience with NPD and treatment history. Participants were asked for their thoughts about communicating with relatives about their result and about whether and how the result was valuable to them. Genetic counseling disclosure sessions were documented in participants' EHR, with detailed three-generation pedigrees that include NPD and other brain disorders; relevant medical articles and healthcare guidelines were also provided to participants' primary healthcare providers.

A mixed-methods approach was utilized to evaluate a sub-set of participants' immediate responses to receiving NPD-related results, including qualitative analyses of written genetic counselor post-disclosure assessments of participant responses and audio recordings of in-person disclosure sessions. Retrospective chart reviews of disclosure session notes were conducted, if needed. The post-disclosure assessments were implemented initially to reduce participant burden and were eventually phased out after this analysis. Audio-recording of in-person disclosure sessions was implemented, with participant written consent (IRB #2017-0273), after results had been reported to the first group of 34 participants and remains on-going. Detailed thematic analysis of a larger dataset of audio-recorded transcripts, which is consistent with this subset, will be reported separately (Wain et al., *in preparation*), but major themes from preliminary analyses are reported here. Post-disclosure assessments were completed for all participants who also consented for audio-recorded sessions. Datasets (audio-recorded transcripts and genetic counselor post-disclosure assessments) were assessed independently using a grounded theory approach[11]. Genetic counselor post-disclosure assessment data were available for both in-person and telephone disclosures (eFigure 1). For each dataset, two independent coders generated codes for themes and subthemes, with

discussion to reach consensus and develop final codebooks. Themes derived from the genetic counselor assessments were also discussed with the entire GSC team.

We took our working definition of "personal utility" from Bunnik et al., 2015 who provide the following: "genomic information has personal utility if and only if it can reasonably be used for decisions, actions or self-understanding which are personal in nature." They go on to clarify that "personal utility can be (indirectly) related to health and disease[12]." This definition informed our interpretation of identified themes which we determined to be related to personal utility.

© 2020 Martin CL et al. *JAMA Psychiatry.*

eReferences.

1. Carey DJ, Fetterolf SN, Davis FD, et al. The Geisinger MyCode community health initiative: an electronic health record-linked biobank for precision medicine research. *Genet Med.* 2016;18:906–13.
2. Kalia SS, Adelman K, Bale S, et al. Recommendations for reporting of secondary findings in clinical exome and genome sequencing, 2016 update (ACMG SF v2.0): a policy statement of the American College of Medical Genetics and Genomics. *Genet Med*. 2017;19(2):249-255. doi: 10.1038/gim.2016.190
3. Dewey FE, Murray MF, Overton JD, et al. Distribution and clinical impact of functional variants in 50,726 whole-exome sequences from the DiscovEHR study. *Science.* 2016;354(6319):aaf6814 https://doi.org/10.1126/science.aaf6814.
4. Staples J, Maxwell EK, Gosalia N, et al. Profiling and Leveraging Relatedness in a Precision Medicine Cohort of 92,455 Exomes. *Am J Hum Genet.* 2018;102:874–89.
5. Li, H. Aligning sequence reads, clone sequences and assembly contigs with BWA-MEM. arXiv e-print (2013).
6. Packer JS, Maxwell EK, O'Dushlaine C, et al. CLAMMS: A scalable algorithm for calling common and rare copy number variants from exome sequencing data. *Bioinformatics*. 2016;32(1):133-135. doi: 10.1093/bioinformatics/btv547 [doi].
7. Riggs ER, Church DM, Hanson K, et al. Towards an evidence-based process for the clinical interpretation of copy number variation. *Clin Genet.* 2012;81(5):403-412.
8. Crawford K, Bracher-Smith M, Owen D, et al. Medical consequences of pathogenic CNVs in adults: analysis of the UK Biobank. *J Med Genet.* 2019;56:131–8.
9. Wang K, Li M, Hadley D, et al. PennCNV: an integrated hidden Markov model designed for high-resolution copy number variation detection in whole-genome SNP genotyping data. *Genome Res.* 2007;17:1665–74.
10. Williams MS, Buchanan AH, Davis FD, et al. Patient-centered precision health in A learning health care system: Geisinger's genomic medicine experience. *Health Aff (Millwood)*. 2018;37(5):757-764. doi: 10.1377/hlthaff.2017.1557 [doi].
11. Timmermans S, Tavory I. Theory Construction in Qualitative Research: From Grounded Theory to Abductive Analysis. *Sociological Theory*. 2012;30(3):167-186. doi: 10.1177/0735275112457914
12. Bunnik EM, Janssens AC, Schermer MH. Personal utility in genomic testing: Is there such a thing? *J Med Ethics.* 2015;41(4):322-326. doi: 10.1136/medethics-2013-101887 [doi].

© 2020 Martin CL et al. *JAMA Psychiatry.*

eTable 1. Genomic Coordinates and Inclusion Criteria for Copy Number Variants

Copy Number Variant	Criteria	Genomic Coordinates (GRCh38/hg38; Mb)
1q21.1 (*GJA5*) del/dup	Size >50% of critical region	chr1:147.11-147.92
1q21.1 (*GJA5* + TAR) del/dup	Size >50% of critical region	chr1:145.63-147.92
3q29 del	Size >50% of critical region	chr3:196.03-197.62
5q35 (*NSD*1) del/dup	Size >50% of critical region	chr5:176.3-177.59
7q11.23 (*ELN*) del/dup	Size >50% of critical region	chr7:73.33-74.73
8p23.1 del/dup	At least 1 Mb of critical region	chr8:8.26-11.91
10q23 del	At least 1 Mb, including *NRG3* and *GRID1*	chr10:79.92-86.98
15q11.2q13.1 BP1-3 del/dup	Full critical region, ~4 Mb	chr15:22.78-28.14
15q13.3 del	Size >50% of critical region	chr15:30.84-32.15
15q24 del	At least 1 Mb between the A-E intervals	chr15:72.67-75.68
16p13.11 del	Size >50% of critical region	chr16:15.42-16.20
16p11.2 distal del	Size >50% of critical region	chr16:28.81-29.04
16p11.2 del/dup	Size >50% of critical region	chr16:29.64-30.19
17p12 (*PMP22*) del/dup	Size >50% of critical region, affecting *PMP22*	chr17:14.19-15.52
17p11.2 (*RAI1*) del/dup	Size >50% of critical region	chr17:16.91-20.30
17q11.2 (*NF1*) del/dup	Size >50% of critical region, affecting *NF1*	chr17:30.78-31.94
17q12 del/dup	Size >50% of critical region	chr17:36.46-37.85
17q21.31 (*KANSL1*) del	Size >50% of critical region	chr17:45.63-46.08
22q11.2 del/dup (proximal, A-B)	Size >50% of critical region	chr22:18.92-20.29
22q11.2 del/dup (proximal, A-D)	Size >50% of critical region	chr22:18.92-21.11
22q11.2 distal del/dup	Size >50% of critical region	chr22:21.44-23.31

Table adapted from Crawford K, Bracher-Smith M, Owen D, Kendall KM, Rees E, Pardiñas AF, *et al.* Medical consequences of pathogenic CNVs in adults: analysis of the UK Biobank. *J Med Genet* 2019;**56**:131–8.

© 2020 Martin CL et al. *JAMA Psychiatry.*

**eTable 2.** ICD-9 and ICD-10 Codes for Neuropsychiatric Disorders, Congenital Malformations, and Chromosomal Abnormalities		
**A. Neuropsychiatric Disorders (NPD)**		
	**ICD-9 Codes**	**ICD-10 Codes**
Intellectual Disabilities	317, 318.0, 318.1, 318.2, 319, 315.5, 315.8	F70, F71, F72, F73, F78, F79, F88
Communication Disorders	315.3, 315.31, 315.32, 315.34, 315.35, 315.39, 307.9	F80.0, F80.1, F80.2, F80.4, F80.8, F80.81, F80.82, F80.89, F80.9,
Autism Spectrum Disorder	299.00, 299.80	F84.*
ADHD	314, 314.0, 314.00, 314.01, 314.1, 314.2, 314.8, 314.9	F90, F90.0, F90.1, F90.2, F90.8, F90.9
Specific Learning Disorder	315.0*, 315.1, 315.2	F81, F81.0, F81.2, F81.8, F81.81, F81.89, F81.9
Motor Disorders	315.4, 307.2, 307.20, 307.21, 307.22, 307.23, 307.3, 781.3	F82, F98.4, F95, F95.0, F95.1, F95.2, F95.8, F95.9, R27.0, R27.8, R27.9
Other Neurodevelopmental Disorders	315.9	F89
Schizophrenia Spectrum and Other Psychotic Disorders	295.*, 297.*, 298.*, 301.22	F20.*, F21, F22, F23, F24, F25.*, F28, F29, F06.1
Bipolar and Related Disorders	296.0*, 296.1*, 296.4*, 296.5*, 296.6*, 296.7, 296.8*	F30.*, F31.*, F34.0
Depressive Disorders	296.2*, 296.3*, 311	F32.*, F33.*, F34.1
Anxiety Disorders	300.0, 300.00, 300.01, 300.02, 300.09, 300.2*, 309.21, 312.23	F40.*, F41.*, F93.0, F94.0
Obsessive-Compulsive and Related Disorders	300.3, 300.7, 312.39	F42.*, F45.22, F63.3
Epilepsy	345.*	G40.*
Cerebral Palsy	343.*	G80.*
*Indicates wildcard character (none or any number)		

**B. Congenital Malformation (CM)**		
	**ICD-9 Codes**	**ICD-10 Codes**
Central Nervous System	740.*, 741.*, 742.*	Q00.*, Q01.*, Q02, Q03.*, Q04.*, Q05.*, Q06.*, Q07.*
Cardiac	745.*, 746.*, 747..0, 747.1, 747.2, 747.3, 747.4*	Q20.*, Q21.*, Q22.*, Q23.*, Q24.*, Q25.*, Q26.*
Cleft lip/palate	749.*	Q35.*, Q36.*, Q37.*
Genital	752.*	Q50.*, Q51.*, Q52.*, Q53, Q53.1*, Q53.0*, Q53.2*, Q54.*, Q55.*, Q56.*
Renal/Urinary	753.*	Q60.*, Q61.*, Q62.*, Q63.*, Q64.*

*Indicates wildcard character (none or any number)

**C. Chromosomal or Structural Abnormality**	
**ICD-9 Codes**	**ICD-10 Codes**
758.*, 795.2, 279.11	Q90.*, Q91.*, Q92.*, Q92.6*, Q93.*, Q95.*, Q96.*, Q97.*, Q98.*, Q99.*, D82.1, R89.8^
*Indicates wildcard character (none or any number); ^Only R89.8 descriptions specifying abnormal genetic test/chromosomal analysis/karyotype were included	

© 2020 Martin CL et al. *JAMA Psychiatry.*

eTable 3. Neuropsychiatric Disorder Diagnoses by Copy Number Variant (Excluding Depression and Anxiety)

Chr	Dosage	CNV	Total	ID	CD	ASD	ADHD	SLD	MD	OND	SCZ	BPD	OCD	EP	CP	Any NPD	Percent Affected
1	Deletion	1q21.1 (*GJA5*)^	59	2	2	0	2	1	0	0	3	4	0	0	0	11	18.6
1	Duplication	1q21.1 (*GJA5*)*	90	2	2	0	3	0	0	0	6	8	0	6	0	16	17.8
3	Deletion	3q29 (*DLG1*)	4	0	0	0	0	0	0	0	0	0	0	0	0	0	0.0
7	Deletion	7q11.23 (*ELN*)	4	1	0	0	1	1	0	0	1	1	0	0	0	3	75.0
7	Duplication	7q11.23 (*ELN*)	8	2	1	0	1	1	0	0	1	1	0	0	0	4	50.0
10	Deletion	10q23 (*BMPR1A*)	1	0	0	0	0	0	0	0	0	0	0	0	0	0	0.0
15	Deletion	15q11.2q13.1 BP1-3 (*UBE3A*)	5	4	1	0	0	1	1	3	0	0	0	1	0	4	80.0
15	Duplication	15q11.2q13.1 BP1-3 (*UBE3A*)	3	1	0	1	0	1	0	0	0	0	0	2	0	3	100.0
15	Deletion	15q13.3 (*CHRNA7*)	55	2	1	1	2	1	1	1	1	1	3	11	0	15	27.3
15	Deletion	15q24 (*SIN3A*)	2	2	0	1	1	0	0	0	0	0	0	0	0	2	100.0
16	Deletion	16p11.2 distal (*SH2B1*)	28	0	2	0	0	0	0	0	0	1	0	0	0	3	10.7
16	Deletion	16p11.2 (*TBX6*)	59	9	2	1	4	2	2	1	0	3	1	5	0	17	28.8
16	Duplication	16p11.2 (*TBX6*)	63	2	1	2	6	3	1	1	4	10	1	4	0	21	33.3
16	Deletion	16p13.11 (*MYH11*)	71	3	4	0	3	1	4	2	2	6	1	18	1	27	38.0
17	Deletion	17p11.2 (*RAI1*)	4	4	2	1	2	0	0	0	0	0	0	3	1	4	100.0
17	Deletion	17p12 (*PMP22*)*	31	0	0	0	0	0	0	0	0	0	0	2	0	2	6.5
17	Duplication	17p12 (*PMP22*)	38	0	1	0	1	0	0	0	2	4	1	2	0	9	23.7
17	Deletion	17q11.2 (*NF1*)	3	0	0	0	1	0	0	0	0	0	0	0	0	1	33.3
17	Duplication	17q11.2 (*NF1*)	4	0	0	0	0	0	0	0	0	0	0	0	0	0	0.0
17	Deletion	17q12 (*HNF1B*)	4	0	0	1	2	1	0	0	1	1	0	1	0	3	75.0
17	Duplication	17q12 (*HNF1B*)	41	1	1	1	4	1	0	0	2	2	1	2	0	11	26.8
22	Deletion	22q11.2 (*TBX1*)	23	13	11	1	6	2	5	2	5	2	0	4	0	17	73.9
22	Duplication	22q11.2 (*TBX1*)^	108	10	6	1	9	2	2	2	5	12	1	3	0	31	28.7
22	Deletion	22q11.2 distal	1	0	0	0	0	0	0	0	0	0	0	0	0	0	0.0
22	Duplication	22q11.2 distal	1	0	0	0	0	0	0	0	0	0	0	0	0	0	0.0
	All CNVs		708	58	37	11	48	18	16	12	33	56	9	64	2	204	28.8

ID: Intellectual Disability; CD: Communication Disorder; ASD: Autism Spectrum Disorder; ADHD: Attention Deficit and Hyperactivity Disorder; SLD: Specific Language Disorder; MD: Motor Disorder; OND: Other Neurodevelopmental Disorder; SCZ: Schizophrenia and Related Disorder; BPD: Bipolar Disorder; OCD: Obsessive Compulsive Disorder; EP: Epilepsy; CP: Cerebral Palsy; CM: Congenital Malformation; NPD: Neuropsychiatric Disorder; * Includes one individual with 1q21.1 Duplication and 17p12 Deletion; ^ Includes one individual with 1q21.1 Deletion and 22q11.2 Duplication.

© 2020 Martin CL et al. *JAMA Psychiatry.*

eTable 4. Congenital Malformation Diagnoses by Copy Number Variant

Chr	Dosage	CNV	Total	Cleft Lip & Palate	Cardiac	CNS	Urinary	Genital	Any CM	Percent Affected
1	Deletion	1q21.1 (*GJA5*)^	59	0	3	2	0	1	6	10.2
1	Duplication	1q21.1 (*GJA5*)*	90	1	8	2	4	1	15	16.7
3	Deletion	3q29 (*DLG1*)	4	0	0	0	0	0	0	0.0
7	Deletion	7q11.23 (*ELN*)	4	0	3	1	0	0	3	75.0
7	Duplication	7q11.23 (*ELN*)	8	0	0	0	0	0	0	0.0
10	Deletion	10q23 (*BMPR1A*)	1	0	0	0	0	0	0	0.0
15	Deletion	15q11.2q13.1 BP1-3 (*UBE3A*)	5	0	1	1	0	3	4	80.0
15	Duplication	15q11.2q13.1 BP1-3 (*UBE3A*)	3	0	0	0	0	0	0	0.0
15	Deletion	15q13.3 (*CHRNA7*)	55	2	1	1	0	2	6	10.9
15	Deletion	15q24 (*SIN3A*)	2	0	0	0	0	0	0	0.0
16	Deletion	16p11.2 distal (*SH2B1*)	28	0	0	0	0	1	1	3.6
16	Deletion	16p11.2 (*TBX6*)	59	0	3	2	1	0	5	8.5
16	Duplication	16p11.2 (*TBX6*)	63	0	3	1	4	1	9	14.3
16	Deletion	16p13.11 (*MYH11*)	71	0	4	0	0	2	6	8.5
17	Deletion	17p11.2 (*RAI1*)	4	0	0	1	0	0	1	25.0
17	Deletion	17p12 (*PMP22*)*	31	0	1	2	0	0	3	9.7
17	Duplication	17p12 (*PMP22*)	38	0	1	1	2	0	4	10.5
17	Deletion	17q11.2 (*NF1*)	3	0	0	0	1	0	1	33.3
17	Duplication	17q11.2 (*NF1*)	4	0	1	0	0	0	1	25.0
17	Deletion	17q12 (*HNF1B*)	4	0	1	0	2	0	2	50.0
17	Duplication	17q12 (*HNF1B*)	41	0	1	1	1	1	3	7.3
22	Deletion	22q11.2 (*TBX1*)	23	3	10	0	1	0	11	47.8
22	Duplication	22q11.2 (*TBX1*)^	108	1	5	1	5	3	13	12.0
22	Deletion	22q11.2 distal	1	0	0	0	0	0	0	0.0
22	Duplication	22q11.2 distal	1	0	0	0	0	0	0	0.0
	All CNVs		708	7	46	16	21	15	94	13.3

CM: Congenital Malformation; CNS: Central Nervous System; NPD: Neuropsychiatric Disorder; * Includes one individual with 1q21.1 Duplication and 17p12 Deletion; ^ Includes one individual with 1q21.1 Deletion and 22q11.2 Duplication.

© 2020 Martin CL et al. *JAMA Psychiatry.*

eTable 5. Combined Congenital Malformation and Neuropsychiatric Diagnoses by Copy Number Variant

Chr	Dosage	CNV	Total	Any NPD	Any CM	Any NPD or CM	Percent Affected (NPD or CM)
1	Deletion	1q21.1 (*GJA5*)^	59	11	6	14	23.7
1	Duplication	1q21.1 (*GJA5*)*	90	16	15	24	26.7
3	Deletion	3q29 (*DLG1*)	4	0	0	0	0.0
7	Deletion	7q11.23 (*ELN*)	4	3	3	4	100.0
7	Duplication	7q11.23 (*ELN*)	8	4	0	4	50.0
10	Deletion	10q23 (*BMPR1A*)	1	0	0	0	0.0
15	Deletion	15q11.2q13.1 BP1-3 (*UBE3A*)	5	4	4	4	80.0
15	Duplication	15q11.2q13.1 BP1-3 (*UBE3A*)	3	3	0	3	100.0
15	Deletion	15q13.3 (*CHRNA7*)	55	15	6	19	34.5
15	Deletion	15q24 (*SIN3A*)	2	2	0	2	100.0
16	Deletion	16p11.2 distal (*SH2B1*)	28	3	1	4	14.3
16	Deletion	16p11.2 (*TBX6*)	59	17	5	20	33.9
16	Duplication	16p11.2 (*TBX6*)	63	21	9	26	41.3
16	Deletion	16p13.11 (*MYH11*)	71	27	6	30	42.3
17	Deletion	17p11.2 (*RAI1*)	4	4	1	4	100.0
17	Deletion	17p12 (*PMP22*)*	31	2	3	4	12.9
17	Duplication	17p12 (*PMP22*)	38	9	4	10	26.3
17	Deletion	17q11.2 (*NF1*)	3	1	1	2	66.7
17	Duplication	17q11.2 (*NF1*)	4	0	1	1	25.0
17	Deletion	17q12 (*HNF1B*)	4	3	2	3	75.0
17	Duplication	17q12 (*HNF1B*)	41	11	3	14	34.1
22	Deletion	22q11.2 (*TBX1*)	23	17	11	19	82.6
22	Duplication	22q11.2 (*TBX1*)^	108	31	13	39	36.1
22	Deletion	22q11.2 distal	1	0	0	0	0.0
22	Duplication	22q11.2 distal	1	0	0	0	0.0
	All CNVs		708	204	94	250	35.3

CM: Congenital Malformation; NPD: Neuropsychiatric Disorder; * Includes one individual with 1q21.1 Duplication and 17p12 Deletion;
^ Includes one individual with 1q21.1 Deletion and 22q11.2 Duplication.

© 2020 Martin CL et al. *JAMA Psychiatry.*

eFigure. Genetic Counselor Results Disclosure Session Outline

Patient: ____________________ MRN: ______________ Date of Visit: _________ GC: _______________

Introductions and Contracting

Thank participant for attending and describe purpose of visit.

Probe: What do you remember about signing up for MyCode?

Explain that a genetic change was identified that can cause medical concerns as well as learning differences and psychiatric illness.

Probe: Tell me about your history of [condition]. What are your beliefs about why you have/had this?

Obtain Family History

Ask for permission to explore family history to get to understand family more fully. Participant may prefer to skip straight to disclosure.

Discuss Results and Assess Response

Provide educational resources and clinical report. Discuss clinical implications and address questions.

Probe: How do you feel about this information? What kind of concerns do you have?

Probe: Does this genetic information change anything about how you understand or view your [condition]? How do you feel about that?

Probe: Does this information change how you think about yourself?

Probe: Who would you talk to about this information? Why/why not?

Probe: How does this experience and receiving this information compare to what you expected?

Follow-up Plan

Review any clinical follow-up with participant and discuss need for additional genetic counselor follow-up for participant or family.

A genetic counselor results disclosure session outline was used to guide sessions, promote consistency between genetic counselors, and to standardize participant response assessment for post-session data analysis.

© 2020 Martin CL et al. *JAMA Psychiatry.*
